# Supplementary material for: A Likelihood Approach for Real-Time Calibration of Stochastic Compartmental Epidemic Models
Source: PLoS Comput Biol. 2017 Jan 17;13(1):e1005257. doi: 10.1371/journal.pcbi.1005257 (PMC5240920; doi:10.1371/journal.pcbi.1005257)
Supplement: S1 File — (TAR.GZ) [file pcbi.1005257.s014.tar.gz › HSPH_Online-SI-Revision/Additions/EnKF08/n100-extreme-SEITR/n100-extreme-SEITR_EnKF2_table-8.pdf]

1	newly 3 weeks spec	2187.	934.82	{125., 549.}	104.731	0.917755	newly 3 weeks cumul	3760.	4537.94	{1083., 4049.}	102.914	0.333602	newly 5 weeks spec	7106.	342.19	{10., 272.}	95.4772	2.03088	newly 5 weeks cumul	14 965.	5443.91	{1346., 4952.}	92.3633	0.793747	newly 59 weeks cumul	91 886.	6213.42	{1520., 7941.}	94.0838	1.50045
2	newly 3 weeks spec	265.	9749.94	{197., 22 534.}	3590.55	1.40515	newly 3 weeks cumul	343.	46 967.4	{16 507., 90 309.}	13 596.1	2.02276	newly 5 weeks spec	3034.	4598.28	{5., 9918.}	102.621	0.740351	newly 5 weeks cumul	4334.	58 318.8	{22 673., 110 207.}	1251.18	1.08185	newly 66 weeks cumul	99 017.	70 508.3	{30 062., 111 811.}	38.47	0.261192
3	newly 3 weeks spec	257.	8326.27	{420., 18 721.}	3151.02	1.34045	newly 3 weeks cumul	379.	42 178.8	{7184., 92 281.}	11 029.	1.87056	newly 5 weeks spec	2684.	5086.91	{3., 12 741.}	166.509	1.07195	newly 5 weeks cumul	3894.	53 664.5	{10 416., 93 686.}	1280.94	1.03154	newly 68 weeks cumul	98 993.	69 980.	{13 436., 99 304.}	30.0568	0.260497
4	newly 3 weeks spec	698.	9606.54	{1047., 20 948.}	1282.95	1.01419	newly 3 weeks cumul	1030.	49 925.3	{9674., 92 434.}	4747.11	1.55662	newly 5 weeks spec	3933.	4298.36	{83., 9457.}	75.6745	0.648061	newly 5 weeks cumul	6733.	60 548.7	{11 886., 104 845.}	802.825	0.873129	newly 66 weeks cumul	96 862.	71 737.1	{28 566., 111 497.}	35.5647	0.243354
5	newly 3 weeks spec	187.	5773.64	{9., 16 108.}	3012.34	1.38404	newly 3 weeks cumul	237.	38 373.9	{2791., 96 083.}	16 094.7	1.9604	newly 5 weeks spec	3231.	3628.68	{0., 11 579.}	123.023	1.49377	newly 5 weeks cumul	4188.	46 483.8	{3784., 96 462.}	1018.81	0.906667	newly 68 weeks cumul	98 880.	58 658.1	{5625., 99 943.}	42.0524	0.449169
6	newly 3 weeks spec	323.	11 525.6	{1319., 20 693.}	3474.44	1.39927	newly 3 weeks cumul	460.	48 916.7	{11 531., 90 584.}	10 534.8	1.91128	newly 5 weeks spec	3690.	5856.34	{21., 12 261.}	113.757	0.781541	newly 5 weeks cumul	5281.	63 169.2	{14 474., 96 884.}	1099.96	1.01816	newly 67 weeks cumul	95 041.	75 854.2	{17 541., 102 318.}	25.6671	0.212498
7	newly 3 weeks spec	2336.	2778.44	{132., 14 255.}	162.584	0.954043	newly 3 weeks cumul	4235.	13 750.5	{936., 69 014.}	307.078	0.48016	newly 5 weeks spec	6848.	593.43	{12., 2063.}	91.6167	1.88142	newly 5 weeks cumul	15 415.	15 635.9	{1150., 76 780.}	139.738	0.803166	newly 59 weeks cumul	97 367.	16 769.2	{1337., 77 825.}	85.9168	1.3579
8	newly 3 weeks spec	476.	9181.66	{1919., 20 881.}	1834.91	1.17081	newly 3 weeks cumul	697.	43 590.9	{8677., 85 924.}	6155.74	1.67394	newly 5 weeks spec	4729.	4423.3	{631., 9110.}	60.3168	0.517945	newly 5 weeks cumul	7052.	54 363.3	{16 179., 96 584.}	673.622	0.816292	newly 66 weeks cumul	99 432.	66 991.	{29 757., 111 756.}	38.6946	0.273637
9	newly 3 weeks spec	88.	5920.26	{153., 15 318.}	6642.68	1.5961	newly 3 weeks cumul	112.	46 260.2	{5674., 96 038.}	41 203.8	2.40039	newly 5 weeks spec	2227.	3833.02	{0., 10 741.}	171.388	1.6324	newly 5 weeks cumul	2748.	54 765.3	{5859., 97 905.}	1896.67	1.15052	newly 68 weeks cumul	99 354.	68 676.4	{6206., 98 568.}	32.1163	0.316617
10	newly 3 weeks spec	982.	5348.72	{877., 10 400.}	450.226	0.561215	newly 3 weeks cumul	1566.	40 034.1	{10 477., 103 056.}	2456.46	1.23213	newly 5 weeks spec	4646.	1501.08	{65., 4074.}	75.2428	0.934731	newly 5 weeks cumul	8317.	44 246.	{11 794., 121 676.}	438.386	0.600488	newly 64 weeks cumul	99 174.	46 670.1	{11 965., 121 856.}	60.4477	0.513659
11	newly 3 weeks spec	578.	2418.68	{0., 8280.}	399.187	1.49782	newly 3 weeks cumul	848.	29 963.8	{6643., 68 152.}	3435.42	1.3884	newly 5 weeks spec	4035.	1037.3	{0., 3407.}	89.2347	2.4499	newly 5 weeks cumul	6425.	32 496.	{6911., 68 608.}	412.775	0.603875	newly 63 weeks cumul	99 600.	35 419.6	{6959., 69 223.}	67.7355	0.649425
12	newly 3 weeks spec	936.	5990.6	{912., 13 448.}	545.558	0.656091	newly 3 weeks cumul	1372.	45 255.6	{13 355., 116 153.}	3199.98	1.37273	newly 5 weeks spec	5911.	1484.4	{72., 3631.}	79.1494	0.985456	newly 5 weeks cumul	9781.	49 581.9	{14 362., 120 617.}	411.259	0.60401	newly 64 weeks cumul	98 924.	51 811.1	{14 392., 120 836.}	55.8696	0.447732
13	newly 3 weeks spec	632.	6957.34	{609., 13 829.}	1009.57	0.869287	newly 3 weeks cumul	864.	66 285.	{25 496., 111 128.}	7571.87	1.79358	newly 5 weeks spec	4789.	1594.11	{3., 4275.}	76.5047	1.20297	newly 5 weeks cumul	7468.	71 098.7	{29 278., 118 299.}	853.95	0.907629	newly 65 weeks cumul	99 507.	72 975.2	{33 554., 119 390.}	37.2539	0.253158
14	newly 3 weeks spec	1910.	1241.88	{139., 4169.}	100.57	0.760767	newly 3 weeks cumul	3449.	7880.03	{2115., 23 323.}	162.035	0.260183	newly 5 weeks spec	5421.	387.63	{2., 1152.}	93.1989	1.88537	newly 5 weeks cumul	12 064.	8932.65	{2490., 28 174.}	93.4135	0.541012	newly 58 weeks cumul	96 176.	9815.51	{2706., 31 005.}	90.2059	1.27023
15	newly 3 weeks spec	1491.	1636.63	{122., 3051.}	142.748	0.763349	newly 3 weeks cumul	2425.	7424.36	{1293., 16 480.}	245.282	0.365335	newly 5 weeks spec	6031.	771.83	{12., 1778.}	95.5807	1.77904	newly 5 weeks cumul	11 560.	9294.01	{1441., 16 977.}	115.196	0.74856	newly 59 weeks cumul	97 278.	10 960.1	{1489., 19 578.}	88.7332	1.47372
16	newly 3 weeks spec	1486.	1221.96	{351., 1823.}	68.179	0.387522	newly 3 weeks cumul	2628.	23 388.8	{9115., 54 496.}	791.328	0.816125	newly 5 weeks spec	4920.	138.85	{2., 274.}	97.1778	2.12119	newly 5 weeks cumul	10 361.	23 921.5	{9141., 57 398.}	138.586	0.27458	newly 62 weeks cumul	92 728.	24 007.5	{9141., 57 821.}	74.3878	0.727268
17	newly 3 weeks spec	187.	7357.6	{691., 19 419.}	3842.55	1.45062	newly 3 weeks cumul	247.	42 307.9	{4709., 95 197.}	17 028.7	2.04366	newly 5 weeks spec	2188.	4594.22	{2., 11 867.}	187.552	1.15303	newly 5 weeks cumul	3077.	52 660.9	{6319., 96 307.}	1612.5	1.10053	newly 68 weeks cumul	93 253.	69 603.1	{7563., 99 500.}	29.9763	0.256031
18	newly 3 weeks spec	162.	12 057.9	{687., 20 697.}	7343.18	1.66443	newly 3 weeks cumul	222.	56 817.5	{15 398., 96 226.}	25 493.5	2.31497	newly 5 weeks spec	2807.	6110.92	{10., 12 046.}	165.716	0.876112	newly 5 weeks cumul	3654.	71 679.5	{31 850., 96 615.}	1863.03	1.23811	newly 67 weeks cumul	99 640.	85 041.3	{54 390., 108 700.}	17.9421	0.132142
19	newly 3 weeks spec	726.	1688.39	{297., 2607.}	172.092	0.337332	newly 3 weeks cumul	1060.	23 316.1	{8545., 69 431.}	2099.64	1.18253	newly 5 weeks spec	4054.	358.84	{6., 288.}	95.1865	1.87395	newly 5 weeks cumul	6766.	24 398.6	{8693., 69 806.}	263.029	0.409986	newly 62 weeks cumul	99 318.	24 927.9	{8701., 69 819.}	75.4239	0.769094
20	newly 3 weeks spec	300.	9690.38	{680., 18 224.}	3137.19	1.32682	newly 3 weeks cumul	402.	51 981.8	{11 248., 98 942.}	12 830.8	1.98238	newly 5 weeks spec	3692.	4830.04	{2., 11 964.}	100.165	0.953415	newly 5 weeks cumul	5182.	63 676.9	{14 561., 102 332.}	1132.35	1.01452	newly 67 weeks cumul	98 887.	74 962.3	{18 723., 106 380.}	28.0601	0.217256
21	newly 3 weeks spec	120.	6034.84	{53., 16 215.}	4948.55	1.47394	newly 3 weeks cumul	157.	38 523.1	{5138., 90 675.}	24 437.	2.14228	newly 5 weeks spec	2417.	4099.28	{0., 11 984.}	165.754	1.44036	newly 5 weeks cumul	3205.	47 539.4	{6572., 95 113.}	1387.58	1.01148	newly 68 weeks cumul	99 034.	63 104.9	{8150., 99 859.}	37.0754	0.346787
22	newly 3 weeks spec	1137.	6614.98	{433., 20 285.}	500.969	0.6663	newly 3 weeks cumul	1829.	42 764.2	{7732., 96 195.}	2243.04	1.17589	newly 5 weeks spec	5958.	1872.41	{83., 4016.}	74.4718	0.909275	newly 5 weeks cumul	10 630.	48 031.8	{8170., 108 015.}	367.641	0.596912	newly 64 weeks cumul	96 878.	51 065.	{8179., 112 060.}	55.5412	0.498901
23	newly 3 weeks spec	1235.	5592.46	{334., 11 140.}	383.456	0.626394	newly 3 weeks cumul	1958.	53 135.9	{5088., 97 003.}	2617.77	1.28814	newly 5 weeks spec	6598.	1452.92	{3., 3918.}	79.2143	1.3643	newly 5 weeks cumul	11 490.	57 363.3	{5294., 104 605.}	414.716	0.690359	newly 65 weeks cumul	88 617.	59 896.7	{10 806., 116 059.}	43.7177	0.352785
24	newly 3 weeks spec	102.	10 319.6	{732., 17 076.}	10 018.7	1.80146	newly 3 weeks cumul	138.	51 891.1	{14 595., 97 325.}	37 502.2	2.47499	newly 5 weeks spec	2027.	5128.63	{3., 10 489.}	202.176	0.968095	newly 5 weeks cumul	2665.	64 347.2	{18 532., 97 352.}	2315.51	1.31317	newly 67 weeks cumul	98 866.	75 755.6	{21 842., 103 644.}	25.4121	0.185824
25	newly 3 weeks spec	917.	2897.28	{0., 8379.}	316.528	1.72207	newly 3 weeks cumul	1410.	37 861.	{7165., 88 134.}	2587.37	1.28031	newly 5 weeks spec	3941.	1109.72	{0., 3915.}	80.7942	2.48928	newly 5 weeks cumul	7263.	40 641.5	{7386., 96 389.}	465.812	0.654232	newly 63 weeks cumul	94 839.	45 192.6	{7470., 96 677.}	57.2334	0.487914
26	newly 3 weeks spec	2138.	581.79	{0., 472.}	102.741	1.78144	newly 3 weeks cumul	3825.	8289.59	{434., 20 584.}	165.363	0.464045	newly 5 weeks spec	6408.	204.91	{0., 85.}	98.0953	3.10811	newly 5 weeks cumul	14 163.	8828.76	{489., 20 617.}	77.6465	0.640013	newly 61 weeks cumul	87 857.	9468.61	{510., 20 715.}	89.5332	1.32584
27	newly 3 weeks spec	1999.	346.73	{0., 468.}	88.5798	1.60178	newly 3 weeks cumul	3536.	6332.67	{732., 17 863.}	123.374	0.395278	newly 5 weeks spec	6511.	95.84	{0., 134.}	98.528	2.99174	newly 5 weeks cumul	13 890.	6592.73	{815., 19 270.}	67.0584	0.585904	newly 61 weeks cumul	98 347.	6822.52	{840., 19 287.}	93.0628	1.37857
28	newly 3 weeks spec	310.	6876.71	{33., 17 535.}	2138.95	1.24693	newly 3 weeks cumul	474.	35 716.7	{5600., 84 266.}	7436.46	1.66454	newly 5 weeks spec	2971.	4375.38	{0., 11 771.}	136.217	1.20354	newly 5 weeks cumul	4384.	45 599.4	{6228., 89 111.}	945.232	0.875263	newly 68 weeks cumul	89 828.	62 241.9	{9476., 100 612.}	35.7037	0.306186
29	newly 3 weeks spec	609.	6641.76	{275., 15 715.}	1007.85	0.886521	newly 3 weeks cumul	951.	65 219.	{29 059., 112 585.}	6757.94	1.74672	newly 5 weeks spec	3642.	1684.89	{1., 4116.}	73.2194	1.27348	newly 5 weeks cumul	6162.	70 158.	{32 667., 120 785.}	1039.69	0.980149	newly 65 weeks cumul	99 052.	72 211.8	{33 966., 126 102.}	39.9302	0.268169
30	newly 3 weeks spec	109.	7782.23	{553., 19 774.}	7050.34	1.65597	newly 3 weeks cumul	138.	40 818.8	{7145., 88 395.}	29 478.8	2.29897	newly 5 weeks spec	1649.	4681.6	{1., 12 570.}	254.95	1.14979	newly 5 weeks cumul	2255.	51 581.9	{9273., 91 615.}	2187.45	1.22768	newly 68 weeks cumul	99 316.	67 423.9	{12 281., 100 340.}	33.7904	0.271872
31	newly 3 weeks spec	3137.	879.11	{74., 1325.}	83.1148	0.901722	newly 3 weeks cumul	6051.	6338.46	{2586., 6179.}	75.3039	0.276899	newly 5 weeks spec	6713.	275.96	{1., 581.}	95.8892	1.99043	newly 5 weeks cumul	17 548.	7089.77	{3055., 6538.}	79.5096	0.611307	newly 58 weeks cumul	87 424.	7617.54	{3282., 10 817.}	91.2867	1.21797
32	newly 3 weeks spec	1418.	5898.04	{1125., 10 065.}	326.817	0.538753	newly 3 weeks cumul	2225.	42 562.	{12 530., 94 823.}	1815.88	1.15357	newly 5 weeks spec	5244.	1668.46	{96., 3590.}	75.8757	0.870353	newly 5 weeks cumul	10 289.	47 231.3	{15 279., 108 312.}	365.328	0.587637	newly 64 weeks cumul	98 867.	50 072.6	{15 311., 112 457.}	56.5523	0.457839
33	newly 3 weeks spec	501.	6420.01	{143., 17 395.}	1206.78	1.0105	newly 3 weeks cumul	673.	41 453.9	{2606., 93 787.}	6063.78	1.51753	newly 5 weeks spec	5187.	3395.3	{0., 10 646.}	84.9389	1.51305	newly 5 weeks cumul	7427.	49 316.3	{3748., 98 391.}	581.638	0.736607	newly 68 weeks cumul	99 003.	63 289.5	{7135., 100 672.}	37.1106	0.376136
34	newly 3 weeks spec	1440.	6475.	{835., 20 302.}	373.049	0.60841	newly 3 weeks cumul	2363.	44 791.6	{7194., 101 935.}	1803.31	1.13577	newly 5 weeks spec	6918.	1976.48	{28., 4449.}	77.3703	1.08086	newly 5 weeks cumul	12 627.	50 213.	{7732., 109 533.}	314.708	0.58087	newly 64 weeks cumul	95 674.	53 603.5	{7737., 114 279.}	54.4064	0.490242
35	newly 3 weeks spec	179.	7136.54	{472., 17 516.}	3899.52	1.41929	newly 3 weeks cumul	229.	37 739.6	{4148., 94 059.}	16 380.2	1.98902	newly 5 weeks spec	2514.	4212.82	{2., 12 236.}	148.246	1.11119	newly 5 weeks cumul	3425.	47 479.8	{4808., 94 116.}	1290.5	0.976851	newly 68 weeks cumul	95 744.	61 670.1	{6442., 100 167.}	39.0712	0.344877
36	newly 3 weeks spec	451.	6439.43	{1131., 17 391.}	1329.06	0.995588	newly 3 weeks cumul	672.	41 782.6	{11 280., 92 468.}	6117.65	1.66548	newly 5 weeks spec	3163.	1644.7	{166., 3527.}	60.9681	0.618124	newly 5 weeks cumul	5077.	46 606.6	{12 878., 104 953.}	818.838	0.848117	newly 64 weeks cumul	99 501.	48 852.9	{12 958., 107 174.}	56.8638	0.444198
37	newly 3 weeks spec	142.	5847.02	{372., 11 116.}	4017.62	1.38646	newly 3 weeks cumul	189.	63 803.5	{25 468., 111 762.}	33 658.5	2.43829	newly 5 weeks spec	2178.	1504.34	{1., 3478.}	75.0523	1.08845	newly 5 weeks cumul	2952.	68 163.2	{29 989., 115 442.}	2209.05	1.28614	newly 65 weeks cumul	99 424.	70 410.5	{33 250., 115 786.}	37.6985	0.253594
38	newly 3 weeks spec	1570.	1410.21	{304., 1838.}	82.2592	0.438902	newly 3 weeks cumul	2591.	21 638.3	{5369., 67 276.}	738.515	0.759813	newly 5 weeks spec	6266.	202.18	{6., 371.}	96.7734	2.10347	newly 5 weeks cumul	12 039.	22 330.8	{7367., 70 054.}	119.108	0.30253	newly 62 weeks cumul	93 543.	22 652.7	{7641., 70 066.}	75.7837	0.793588
39	newly 3 weeks spec	955.	5959.24	{861., 15 544.}	531.359	0.664727	newly 3 weeks cumul	1540.	43 995.4	{11 690., 97 170.}	2757.42	1.30111	newly 5 weeks spec	4980.	1410.71	{68., 3540.}	76.5074	0.97371	newly 5 weeks cumul	8707.	48 210.4	{12 637., 108 919.}	459.099	0.633853	newly 64 weeks cumul	97 320.	50 140.4	{13 307., 110 688.}	55.4135	0.456671
40	newly 3 weeks spec	1152.	6877.33	{556., 12 540.}	517.949	0.74023	newly 3 weeks cumul	1821.	54 528.3	{19 996., 108 381.}	2894.42	1.40494	newly 5 weeks spec	6088.	2217.24	{3., 4188.}	78.0851	1.22724	newly 5 weeks cumul	10 814.	60 577.8	{25 739., 108 566.}	460.799	0.687269	newly 65 weeks cumul	99 384.	64 265.2	{29 027., 117 518.}	42.7879	0.280838
41	newly 3 weeks spec	84.	6483.1	{178., 15 941.}	7633.64	1.70892	newly 3 weeks cumul	118.	44 027.8	{5724., 94 742.}	37 213.1	2.36074	newly 5 weeks spec	1333.	3569.53	{0., 10 573.}	256.61	1.34057	newly 5 weeks cumul	1781.	52 213.7	{6823., 96 654.}	2835.	1.32511	newly 68 weeks cumul	96 330.	64 319.3	{8111., 99 434.}	34.9383	0.333278
42	newly 3 weeks spec	2385.	1387.87	{139., 2056.}	114.525	0.93127	newly 3 weeks cumul	4744.	6406.74	{1249., 4825.}	140.212	0.474129	newly 5 weeks spec	6151.	436.9	{12., 1688.}	93.6319	1.80813	newly 5 weeks cumul	14 873.	7595.97	{1488., 9932.}	103.211	0.827037	newly 59 weeks cumul	96 441.	8785.31	{1549., 25 795.}	91.5163	1.50033
43	newly 3 weeks spec	942.	7342.8	{811., 20 708.}	690.461	0.760349	newly 3 weeks cumul	1522.	43 054.5	{11 175., 95 788.}	2731.16	1.29122	newly 5 weeks spec	4281.	2124.32	{67., 3833.}	64.7652	0.70732	newly 5 weeks cumul	7799.	48 995.2	{11 767., 107 339.}	539.22	0.719004	newly 64 weeks cumul	98 631.	52 585.9	{11 870., 111 296.}	55.1069	0.47218
44	newly 3 weeks spec	717.	6661.76	{920., 15 230.}	830.488	0.777992	newly 3 weeks cumul	1097.	38 858.3	{9465., 92 016.}	3442.24	1.39651	newly 5 weeks spec	5449.	2175.78	{97., 4449.}	74.5183	0.806821	newly 5 weeks cumul	8791.	44 736.4	{10 067., 108 853.}	416.087	0.606623	newly 64 weeks cumul	99 299.	48 575.5	{10 192., 112 928.}	58.5003	0.479689
45	newly 3 weeks spec	514.	10 680.9	{2473., 20 908.}	1984.88	1.22935	newly 3 weeks cumul	734.	50 799.8	{16 636., 87 474.}	6822.86	1.75381	newly 5 weeks spec	4552.	4867.43	{61., 9881.}	67.0833	0.585417	newly 5 weeks cumul	6924.	62 841.8	{22 523., 100 470.}	811.483	0.919124	newly 66 weeks cumul	99 357.	75 177.7	{31 775., 109 394.}	31.0643	0.20188
46	newly 3 weeks spec	247.	6027.17	{59., 17 307.}	2360.91	1.28586	newly 3 weeks cumul	335.	38 955.9	{4278., 93 041.}	11 530.4	1.84168	newly 5 weeks spec	2717.	3546.18	{0., 10 866.}	128.374	1.30424	newly 5 weeks cumul	3855.	47 055.	{5224., 95 550.}	1127.7	0.95074	newly 68 weeks cumul	99 095.	58 608.3	{5884., 97 946.}	42.9833	0.432953
47	newly 3 weeks spec	977.	2272.28	{0., 5859.}	245.797	1.54927	newly 3 weeks cumul	1597.	30 044.4	{7024., 69 001.}	1783.76	1.14073	newly 5 weeks spec	4864.	925.64	{0., 3043.}	89.1423	2.87399	newly 5 weeks cumul	8755.	32 389.8	{7338., 69 165.}	281.018	0.504236	newly 63 weeks cumul	96 045.	34 626.7	{7352., 69 165.}	67.1779	0.610296
48	newly 3 weeks spec	364.	10 477.3	{2255., 21 507.}	2782.02	1.31257	newly 3 weeks cumul	527.	53 668.3	{18 499., 111 383.}	10 084.	1.87218	newly 5 weeks spec	3183.	4791.91	{237., 10 969.}	102.57	0.565318	newly 5 weeks cumul	4879.	65 487.7	{23 690., 123 398.}	1246.96	1.06278	newly 66 weeks cumul	97 052.	77 159.4	{31 730., 124 571.}	35.4815	0.241535
49	newly 3 weeks spec	1900.	6702.69	{965., 20 576.}	281.926	0.532589	newly 3 weeks cumul	3162.	50 406.2	{8971., 119 942.}	1495.83	1.03844	newly 5 weeks spec	7611.	1716.04	{46., 3694.}	81.8783	1.22231	newly 5 weeks cumul	14 764.	55 378.6	{10 454., 122 856.}	288.376	0.503155	newly 64 weeks cumul	95 259.	58 190.4	{10 454., 123 084.}	52.2657	0.423375
50	newly 3 weeks spec	943.	5995.53	{365., 11 177.}	559.081	0.740636	newly 3 weeks cumul	1533.	54 135.1	{10 673., 110 072.}	3436.17	1.43299	newly 5 weeks spec	5024.	1497.65	{2., 3278.}	73.9791	1.23426	newly 5 weeks cumul	8966.	58 586.3	{14 579., 114 022.}	563.735	0.792076	newly 65 weeks cumul	98 082.	60 606.8	{18 256., 116 712.}	48.4504	0.402346
